# Experience of primary dental care teams in managing the oral health of oncology patients

**DOI:** 10.1186/s12903-024-05203-8

**Published:** 2024-12-26

**Authors:** Callum Wemyss, Ahmed Abdulsalam, Laura Beaton, Kirsten Perry, Claire Scott, Douglas Stirling, Michele West

**Affiliations:** 1 NHS Education for Scotland, Frankland Building, Small’s Wynd, Dundee, DD1 4HN UK; 2 School of Dentistry, University of Dundee, Small’s Wynd, Dundee, DD1 4HN UK

**Keywords:** Oral health, Cancer, Primary care dentistry, Oncology

## Abstract

**Background:**

Estimates suggest that one in two people will experience cancer in their lifetime. Cancer and the treatment of cancer can have several impacts on oral health. It is therefore important that dental teams are supported in managing this group of patients especially in primary care dental settings, where most of these patients will first present to dental services. The aim of this study was to explore current practice and beliefs about managing patients with, or who have had, cancer in primary dental care settings.

**Methods:**

Online focus groups consisting of dental professionals working in primary care dental settings in Scotland, were conducted. Areas explored included cancer types seen, perceived role, challenges, and areas where further support was desired. Data from focus group transcripts were analysed using thematic analysis.

**Results:**

Four focus groups were conducted with a total of fifteen participants. Themes identified related to the types of cancers seen in primary care dental settings; communication between dental and medical teams; patient experience; mixed healthcare messages; patient engagement with their healthcare; challenges in treatment planning; apprehension about what can safety managed in primary care; and wider system factors influencing the management of patients with, or who have had, cancer. Areas where support exists but further support is desired were also identified.

**Conclusions:**

Challenges appear to exist in the provision of oral healthcare for patients with, or who have had, cancer. This study has indicated several areas where further support could be targeted. The results should be validated by further research.

**Supplementary Information:**

The online version contains supplementary material available at 10.1186/s12903-024-05203-8.

## Background

In 2021, Scotland recorded 35,379 cancer cases, a 5.5% increase since 2019 [1]. The number of cancer survivors in developed countries is also rising, thanks to earlier diagnoses and tailored treatment plans [2]. Combined with an aging population, this suggests an anticipated increase in the number of patients with, or who have had, cancer accessing primary care dental services. Cancer treatments continue to advance, encompassing modalities such as surgery, cytotoxic chemotherapy, targeted therapy (including immunotherapy), radiotherapy, hormonal therapy, and various types of bone marrow transplantation. When dentists treat patients with cancer or who have previously been treated for cancer, it is crucial to understand the effects of these treatments on oral health to provide comprehensive oral care. The side effects of cancer treatment on oral health are well documented and include conditions such as oral mucositis, oral infections, xerostomia, trismus, osteonecrosis, taste changes, and ulceration. Additionally, cancer patients have a higher risk of developing dental caries. These oral health complications can significantly impact an individual’s overall wellbeing [3]. 

Patients with a history of cancer or those currently undergoing cancer treatment may present to the primary care dental team either electively or upon referral by a member of their multidisciplinary oncology team. These visits can occur at different stages: before, during, or after cancer treatment. Each stage of the cancer treatment journey presents specific roles and challenges for the primary care dental team. Examples of these roles and challenges at each stage are outlined in Table 1.

**Table 1:** Stages of cancer treatment and related roles of primary dental care team [4–7]

Stage of treatment	Role of primary dental care team
♣ *Prior to surgical*,* systemic anti-cancer therapy* ♣ *or radiological intervention*	• Providing prevention and information about the risks of cancer treatment on oral health. • Eliminating any oral sources of infection to avoid risk of systemic spread (e.g. sepsis) • Eliminating any teeth of particularly poor prognosis in an attempt to reduce the need for extractions or minor oral surgery in the future in situations where the patient may be at risk of: • Medication related osteonecrosis of the jaw (MRONJ) • Osteoradionecrosis (ORN)
♣ *During systemic anti-cancer therapy or radiological intervention*	• Addressing acute problems including dental pain and infection. • Management of dry mouth or oral mucositis. • Managing oral manifestations of bacterial, fungal and viral infections due to increased risk of infection. • Consideration of increased bleeding and infection risk if treatment is being carried out. • Ongoing prevention
♣ *After surgical*,* systemic anti-cancer therapy or radiological treatment*	• Prevention of dental disease • Managing reduced mouth opening (trismus due to effect of radiotherapy on muscles and other soft tissues can cause problems with access to oral cavity for self-performed plaque control and executing dental treatment) • Management of dry mouth and associated oral soreness. • Management of patients at risk of MRONJ or ORN who need dental treatment. • Diagnosis and referral of patients with MRONJ or ORN. • Head and neck cancer surveillance - particularly for patients who have had oral or oropharyngeal cancer. • Consideration of laryngectomies or tracheostomies and any dietary considerations. • Maintenance of prosthodontic rehabilitations including implant supported or retained prostheses.

To address the challenges faced by primary dental care teams and improve the quality of patient care, various guidelines have been developed on management and maintaining the oral health of patients with a history of cancer. The Royal College of Surgeons (England) and the British Society for Disability and Oral Health published clinical guidelines in 2018 titled “Oral Management of Oncology Patients Requiring Radiotherapy, Chemotherapy, and/or Bone Marrow Transplantation” [4]. These guidelines offer an overview and evidence-based recommendations for dental management of cancer patients before, during, and after treatment.

Similarly, the Specialist Pharmacy Service in NHS England issued advice primarily aimed at general dental practitioners in primary dental care, titled “How Should Adults with Cancer Be Managed by General Dental Practitioners if They Need Dental Treatment?” [8]. For patients with cancers that can metastasize to bones, anti-resorptive or anti-angiogenic drugs may be prescribed to prevent bone fractures and for prevention of cancer recurrence. Patients taking these medications are at risk of developing Medication-related Osteonecrosis of the Jaw (MRONJ) with recent reports estimating an incidence of less than 5% [9]. The Scottish Dental Clinical Effectiveness Programme (SDCEP) published guidance titled “Oral Health Management of Patients at Risk of Medication-related Osteonecrosis of the Jaw” (2017), which offers key recommendations for the dental team on management of patients at risk of MRONJ [10]. In the United Kingdom (UK), it is recommended that head and neck cancer multidisciplinary teams (MDT) should include a restorative dentist and patient assessment should be carried out pre and post treatment in a service led by a consultant in restorative dentistry [11, 12]. Standards also recommend that patients should have access to a suitably experienced dental therapist or hygienist [12]. Once specialist dental care is no longer required, patients will be discharged back to their primary dental care team with a long-term management plan to receive ongoing care and monitoring [5]. No similar recommendations have been made for MDTs caring for patients with other types of cancer. To our knowledge, there is limited evidence exploring the experiences of primary dental care teams on managing patients with, or who have had, cancer which is important to help identify areas that require attention to better support primary dental care teams when managing this group of patients.

The primary aim of this study was to explore current practice and beliefs about managing patients with, or who have had, cancer in primary dental care which will inform the need for, and scope of clinical guidance targeted at primary care dentists on the oral health management of patients with, or who have had, cancer.

## Methods

A qualitative design using online focus groups was selected as the most appropriate way to meet the aims of the study. Focus groups were chosen as an efficient way of collecting data while also allowing broad discussions of topics in an area not previously explored in the literature. Online participation was deemed to provide busy dental team members, as well as those who are working in remote areas of Scotland, a greater opportunity to participate. This study has been reported using Consolidated Criteria for Reporting Qualitative Research [13]. 

### Sampling and recruitment

A convenience sampling strategy was used by sending out email invitations to all dental professionals (DPs) in Scotland who held an NHS Education for Scotland (NES) Portal Account. Dental professionals include both dentists and dental care professionals (e.g. dental hygienists, dental therapists, dental nurses) [14]. NES Portal is an online tool used for course bookings/ management administered by NES. Only those who had previously opted in to receive marketing correspondence were invited. Recruitment advertisements were added to the social media accounts of the NES Dental Directorate and to the NES Clinical Effectiveness website. Those sampled were provided with a privacy statement and participant information sheet which included eligibility criteria (Table 2). Individuals who expressed an interest in participating were asked to provide demographic information, their job role and availability using an online form. It was made clear that participation was voluntary.

**Table 2:** Inclusion and exclusion criteria

Inclusion criteria	Exclusion criteria
Registered dentists or registered DCPs working in primary care in Scotland	Dentists or DCPs working in secondary care / hospital dental service
Any demographic or protected characteristic	Dentists or DCPs not registered to work in UK
Dentists and DCPs working in the Scottish Public Dental Service who provide care under general dental service regulations	Dentists or DCPs not currently providing care in Scotland

DCP Dental Care professionals (Dental therapists, hygienists, nurses, clinical dental technicians)

Forty-two DPs expressed an interest in participating and 23 were invited to participate (based on eligibility, and availability of both the research team and potential participants). Recruitment for the focus groups ended when data saturation was reached. Focus groups were organised with the aim of approximately six participants per group [15]. This number was chosen as it provides everyone an opportunity to contribute yet at the same time provides a diverse spread of experiences and thoughts. The make-up of each focus group was informed by the researchers to ensure a satisfactory mix of clinical experiences and backgrounds. Dentists working in the public dental service (PDS) and general dental services (GDS) were separated into different groups; this was because dentists in the PDS will often provide oral health care on referral from dentists working in GDS. It was expected that the two groups would have different experiences and that separating these participants would facilitate the effectiveness of discussions.

### Data Collection

Before convening each focus group, the research team ensured that all participants were provided with a participant information sheet (PIS) and had completed an online consent form. The focus groups were convened using *Microsoft Teams* and moderated by one member of the research team (CW). CW is an oral surgery trainee with prior experience and training in qualitative methods. Another member of the research team (AA), a dental core trainee developing his qualitative research experience, asked a selection of the questions. There was an experienced qualitative researcher (LB or CS) present at each focus group. Participants were made aware of the researchers’ backgrounds in the introductions as well as the rationale for the research. Based on the project aim and a review of available guidance on the topic, a questioning route was formulated by the research team (Supplementary Material 1). It comprised the following topics:

- Common cancers seen in primary dental care settings.
- Role of the primary care dental team.
- Challenges and barriers.
- Gaps in knowledge.
- Support currently available.
- Aspects to include if specific guidance was developed.

The questioning route was piloted on two dentists working in primary care and subsequently adapted and refined following reflection and feedback. Data from the pilot focus group were not included in the analysis.

Video and audio recordings of the focus groups were facilitated using the recording feature on *Microsoft Teams*.

### Analysis

Focus groups were transcribed by a member of the research team’s business support team, using the automatically generated transcription provided by *Microsoft Teams* as a starting point. Focus group transcripts were manually coded by the research team using the thematic analysis method outlined by Braun & Clarke, 2006 [16]. Initially a sample of one transcript was coded by four members of the research team (CW, AA, LB and CS) and a calibration exercise was undertaken. The remainder of this transcript was coded by CW and AA before further calibration took place between AA and CW. The remainder of the initial coding was divided between CW and AA. A coding key was developed, themes and subthemes were identified and agreed by CW and AA. Selecting themes and sub themes was an iterative process facilitated by research team meetings (CW, AA, LB and CS) until a consensus was reached. Themes and sub themes were confirmed by cross checking with the data set and associated data extracts.

### Ethics and Governance

This work is categorised as service development; therefore, NHS Ethics or research and development approval was not required. This was confirmed by using the NHS Health Research Authority Decision tool [17]. This work was conducted in accordance with the UK Policy Framework for Health and Social Care Research and the 2021 edition of the Governance Arrangements for Research Ethics Committees (NHS Health Research Authority) [18, 19]. 

## Results

Four online focus groups, involving fifteen participants were convened. Characteristics of the Dental Professionals (DPs) and make-up of the focus groups is detailed in Table 3. Eight of the fourteen Scottish territorial health boards were represented in the focus groups. Focus groups ranged from 44 to 75 min with an average time of 59 min. The final number of participants in each group varied due to loss of DPs who did not attend focus groups or circumstances where it was not possible to substitute late dropouts due to other DPs’ availability and time.

**Table 3:** Characteristics of participants and make-up of focus groups (*n* = 15)

Focus group	Participant	Gender	Professional group	Other notes	Dental service
Focus Group 1	DP1	Female	Dentist	Principal Dentist	GDS
	DP2	Female	Dentist	Principal Dentist	GDS
	DP3	Female	Dental Therapist	Previous experience in PDS	GDS
Focus Group 2	DP4	Female	Dentist	Specialist in Special Care Dentistry	PDS
	DP5	Male	Dentist	Specialist in Special Care Dentistry	PDS
	DP6	Female	Dentist		PDS
	DP7	Female	Dentist		PDS
	DP8	Female	Dentist		PDS
	DP9	Male	Dentist		PDS
	DP10	Male	Dentist		PDS
Focus Group 3	DP11	Female	Dentist		GDS
	DP12	Male	Dentist		GDS
Focus Group 4	DP13	Female	Dentist		GDS
	DP14	Female	Dental Therapist		PDS
	DP15	Female	Dental Hygienist		PDS

GDS general dental service, PDS Public dental service

Ten themes were identified with several sub themes (Table 4).

**Table 4:** Themes and sub-themes from focus groups

Theme	Sub-themes
Mix of patients with different cancers seen	Most common cancers seen
	Head and Neck seen rarely in GDS
	PDS cohort
	Paediatrics seldom seen in primary care
Dental teams working in primary care are clear in their roles	Managing and maintaining oral health
	Dental input through cancer journey
	Willingness to see these patients
	Pastoral
	Signposting
Mixed healthcare messages	Oral health
	When to visit dentist
Communication between dental and medical teams	Variation in effectiveness of communication
	Integration
	Social Attitudes
Patient experience	Physical
	Mental
Patient engagement with their healthcare	Poor attitudes to oral health
	Lack of knowledge about their cancer management
	Proactive patients
Treatment planning challenges	Chaotic
	Managing expectations
	Pre-existing oral health
	Different cancer treatments/difference between oral and other cancers
	Prevention advice
Apprehension from dental teams	Fear
	Knowledge
	Complexity
Wider system factors influence management in primary care	Organisational secondary care
	Payment system
	Referral pathways
	Access to general dental practitioners
Support	Guidance
	Patient information and other accompanying resources
	Suggestions for topics to include
	Supportive colleagues

### Mix of patients with different cancers seen

Participants had experienced caring for patients with a variety of different cancers. The most common appeared to be breast and prostate cancer. Some participants working in the PDS saw a frequent stream of haematological cancer types such as lymphomas, leukaemia, myelomas. Dental teams working in some boards were also involved in the head and neck cancer multidisciplinary team. Other cancers mentioned included stomach, bowel, lung and skin. DPs in both the PDS and GDS reported to have cared for patients who were receiving palliative care. Head and neck cancer was rarely seen in GDS with dental teams only able to recall a handful of patients they had cared for over their practising careers. Both participants in PDS and GDS did not recall any experience of caring for children with cancer and it was suggested that these patients were probably seen by dental services in secondary care.

### Dental teams working in primary care are clear in their roles

Dental teams were asked to explain their role in the management of oncology patients. Team members were clear about their role including managing and maintaining the oral health of their patients, whether this be pre-, mid- or post cancer treatment.

*“… mainly the managing the after effects*,* the patients are living on with a fairly difficult standard of life*,* immunocompromised and dry mouth*,* that sort of thing. So just a lot of prevention and a lot of trying to maintain dentitions without anything too fancy. Just keep them going so that they can have a reasonable standard of life”. [DP10]*

It was felt that all members of the team, including dental therapists, hygienists and dental nurses, had a role in the management of these patients.

*“… dental nurses are certainly often involved with communication*,* between us and the*,* you know*,* the patient. A lot of them are dental nurses will deal with patients on the reception*,* so they’re often the first port of call actually”. [DP12]*

Dentist participants also cited the superior experience of dental therapists in providing oral hygiene instruction.

A proactivity and willingness to see these patients came across in all focus groups.

*“… we’d want to see these patients more frequently rather than less frequently*,* you know*,* want to see them more often to help them to give them advice and to prevent problems rather than don’t*,* don’t come”. [DP2]*

*“… we will bust a gut to get them in and make sure that they are definitely fit before they start their treatment”. [DP1]*

The pastoral and supportive role dental team members have for their patients during a very emotional time of their lives was recognised, especially in the context that many DPs may already have longstanding relationships with their patients.

*“… it’s a very*,* very traumatic time for them. I will get them in. I will do anything I can to get them in so we can see them because that just lessens their worry and makes our lives a lot easier in the long run and they know we’re always at the end of the phone*,* the number of times I do get phone calls saying*,* can I speak to you about whatever? So I do speak to them … because we see them more regular than their GPs*,* you know*,* we’re the ones that they will call more than anything else”. [DP1]*

There was a recognition that there was only so much that dental teams could provide when dealing with some of these emotional and functional issues, but participants were clear on their role of signposting.

*“… a lot of it was reassurance*,* signposting to other people quite often after*,* if they were having issues with their diet or swallowing erm*,* because they would often quite open up to you and they’re just trying to signpost to other people”. [DP14]*

### Mixed healthcare messages

Mixed messages on attending the dentist during cancer treatment and oral health were also raised by participants. Some had experienced situations where patients had been advised by oncology teams not to attend dental appointments before or during their treatment.

*“Yeah*,* they often say they’ve almost been told not to come to us*,* erm*,* because of the risks associated with having treatment during … I mean they probably*,* you know it would be fine to have a scale and polish or fluoride varnish applied or whatever”. [DP13]*

*“… if the oncologist and the*,* you know*,* oncology nurse are putting the fear of God in them*,* don’t go and see your dentist*,* absolutely don’t*,* and then something goes wrong and they need to come and see us*,* but they don’t want to come and see us because they’ve been told not to come and see us*,* it’s not helpful”. [DP2]*

Mixed messages on oral hygiene advice were also discussed by more than one focus group, with the advice provided by medical teams, e.g. to use a soft toothbrush, being contested by DPs.

*“… one of my pet hates is when the oncology nurse says use a soft toothbrush*,* and I’m going*,* surely minimising any source of infection*,* whether it be gingivitis is our priority*,* and trying to explain to them to maintain their oral hygiene…”. [DP1]*

*“We find a lot of the patients are just told automatically to use a soft toothbrush*,* but then it doesn’t move plaque so well and they end up with worse*,* worse problems than if they just continued using the toothbrush they were using”. [DP4]*

Some participants felt that some of the mixed messages around dental attendance during cancer treatment originated partly from members of the oncology or medical team.

*“I think it is probably kind of (an) urban myth coupled with the oncology*,* the oncology nurses*,* the older school ones*,* have maybe said*,* I think that’s where it’s coming from”. [DP1]*

### Communication between dental and medical teams

There was variation in the experience of dental teams regarding communication with oncology teams. In some situations, communication appeared to be effective, with oncology teams very supportive of dental teams. This appeared to vary with location and the dental service the DP worked in.

*“And in terms of colleagues*,* we’ve got clinical nurse specialists an invaluable resource*,* and we’re lucky to have direct access to consultants as well who are very good and prompt at responding to emails or answering the phone and other things”. [DP5]*

Difficulties in communication were also frequently cited. Some participants explained that patients would contact the dentist explaining they needed an oral health assessment prior to their cancer treatment but accompanied by no formal lines of communication from the oncology team.

*“It’s mainly the patient that comes and says eh*,* my doctor has told me to come to have this dental check-up to do this treatment*,* but it’s it hasn’t been like a formal letter*,* so that would be good to like to try to have more communication between the oncologist and the dental practice”. [DP12]*

There was some discussion around the lack of integration and importance placed on oral health during cancer treatment and the attitudes of medical teams.

*“Often feel that dentistry is a bit of an afterthought when it comes to the cancer”. [DP12]*

*“… it’s not integrated … I feel that we’re all clinicians all in this together*,* that it should be more joined up with all of the medical team*,* … I just think that we need to be even better connected to dietetics and better connected with speech and language and everything else”. [DP3]*

This, compounded with situations when patients do not know information about their own care or where there is a lack of information on patient prognosis, can cause further difficulty.

*“Yeah*,* I mean try to [contact the patient’s medical teams]. It’s quite difficult though patients often don’t really remember the names or the contact details*,* and it can be quite difficult to track down”. [DP2]*

*“… we don’t have a huge idea of prognosis*,* you know or they expect it to have curative*,* curative treatment? Or is it going to be palliative? I think that could really quite warp treatment plans with are we going to be actually quite aggressive”. [DP8]*

### Patient experience

Participants discussed both physical and mental aspects influencing their patients’ experience and quality of life while on care pathways. Treatment side effects can cause many physical problems for patients and those with head and neck cancer have site specific side effects which can be particularly debilitating and associated with risks.

*“I’ve had a few patients who have had previous tracheostomies and are now nil by mouth*,* not allowed to swallow*,* can’t have any fluids*,* all peg fed and there’s been quite a lot of challenges with aspiration for those patients”. [DP8]*

As noted above, participants outlined how emotions from patients and their families may manifest at visits to dental teams.

*“… just recently*,* you know*,* a wife actually just crying in the surgery about her husband*,* who was in for treatment. … Patients telling you things sometimes that are non-dental but are obviously really worrying them or concerning them . about their general health sometimes after treatment*,* the worrying about maybe even cancer coming back*,* especially oral cancer …the patient will just break down in the surgery or just feel that you’re the person at that moment they*,* they can tell or speak to”. [DP15]*

*“… another patient that had quite recently been diagnosed with breast cancer and she just come in for a check-up and it was quite a difficult conversation. She got very upset*,* you know*,* when I asked her about her medical history and*,* you know*,* that that was … quite a …. difficult consultation”. [DP2]*

Some participants explained how these patients will often present with general healthcare anxiety and some will also have specific dental anxieties exacerbated by the mental aspects of dealing with cancer.

*“So they’ve been referred into us and often they’re just anxious about all of the treatment they’re getting*,* including their dental treatment. So*,* it’s just about that whole thing about getting them in and just allowing them to have a chat with us and feeling quite comfortable before we start giving them all the advice”. [DP15]*

The volume of information patients will be provided with during their cancer journey was recognised.

*“… patients they’re overwhelmed already with all the information with everything has been going on for them they are taking everything in and then we are focusing very much on teeth and potentially quite a lot of complex discussions in that”. [DP9]*

*“Speaking through side effects cause quite often there’s a lot of information they get*,* so they do often when they see you*,* have a lot of questions or can’t remember many things … They found out their cancer and then went straight to dental so it was just a lot of information”. [DP14]*

### Patient engagement with their healthcare

The role of patients was also discussed by participants. It was recognised that in some cases patients with cancer may have poor pre-existing attitudes to their oral health.

*“… they’ve avoided dentistry for many years and it’s the cancer diagnosis that’s forced them to address those issues”. [DP5]*

Where oral health sits in terms of other priorities for the patient was also raised.

*“Obviously*,* it’s really hard. It’s not their priority quite often. Head and neck was a bit different because that was the area*,* but if it was like breast or haematological it was trying to motivate them for oral health as well as their general health could be quite difficult”. [DP14]*

A subtheme that emerged in many of the focus groups was around patient knowledge and understanding about their cancer diagnosis and treatment.

*“… quite often the patient will come in and they can’t remember*,* they don’t know exactly what kind of treatment they’re having”. [DP2]*

However, there was evidence provided by some participants that these were not observations seen in all patients with cancer with some having a more proactive approach to their healthcare.

*“Well*,* the patients are very good at our practice of bringing letter to say they are about to start their treatment… Yeah*,* there’s a lot who will come in and say I’m due to start my chemo very soon”. [DP1]*

### Treatment planning challenges

Some of the challenges discussed above also contributed to challenges in treatment planning for these patients, particularly in the pre-cancer treatment stage. Poor pre-existing oral health was cited as a challenge often resulting in the need for more treatment.

*“I’ve got eight rotten teeth in my mouth’*,* you know. Sometimes people leave it that long to come in erm or*,* or they’ll come asking you to sign a letter that*,* that says they’re dentally fit”. [DP13]*

*“… it’s not uncommon that patients are coming to us with really poor*,* neglected dentitions”. [DP5]*

Depending on the cancer treatment being provided, the recommended treatment will sometimes be quite aggressive (i.e. removing several teeth) and managing patient expectations in the context of also being recently diagnosed with cancer makes these conversations more difficult.

*“And then next thing they know we’re telling them they need all this stuff taken out again [teeth removed or crown and bridgework dismantled]*,* and they’re gonna be stuck with a denture that they’re not gonna be happy with and been trying to avoid”. [DP9]*

Making decisions on the prognosis and treatment planning of teeth with questionable prognosis was mentioned several times. DPs explained the pressure they faced making decisions on teeth with questionable or uncertain prognoses. Teeth with a poor periodontal or endodontic condition were given as examples. DPs also raised a situation which occurs frequently where patients will present with very heavily restored dentitions where they have been provided with multiple crowns and/or bridgework. This work may have been in place for many years not causing problems however depending on the quality of the work, may have the potential to cause problems in the future. DPs explained that it can be difficult for their patients to come to terms with the possibility that this dental work may need undone.

There was recognition given to the fact that each case should be taken on its own merit and the treatment plan should be bespoke for the patient.

*“But*,* but it’s such a broad*,* it’s such a broad thing cancer*,* that you know and the treatments are so different*,* you know people some people are really ill with their cancer treatment and some people just seem to go on working and*,* and doing everything they normally do”. [DP13]*

It was clear that the pre-cancer treatment stage can be quite chaotic with short timescales to complete any necessary dental treatment before the cancer treatment.

*“I would say time frame is probably the biggest issue*,* but in particular again going back to the head and neck MDT*,* we go to the meeting in the morning*,* the patients are usually seen early afternoon by the oncologist plus or minus the surgeon and then they just basically just rock up to our department in the afternoon at any time”. [DP4]*

*“I would say time frame is often very challenging because we’ll get a referral and they’ll be wanting to start chemo the next week and or start the bisphosphonates as soon as possible*,* and you know we often have a mouthful of neglect that needs multiple teeth taken out*,* needs healing”. [DP7]*

### Apprehension from dental teams

Participants frequently discussed apprehension about what can be safely managed in primary care as a ‘challenge’, and some described this a ‘fear’ when treating certain oncology patients. The fear was often based on the risk of infection or bleeding when treating an oncology patient and often cited as coming from previous training. Examples were given from the act of providing an examination all the way to dental treatments such as extractions.

*“Yes*,* knowing when they’re they are fit enough and there I say*,* can we wield a perio probe appropriately in their mouths to check and that*,* I think that’s the fear of God that’s been put into us during our training is don’t probe*,* don’t do this*,* don’t do that*,* and you’re thinking*,* well how can I actually do a proper diagnosis unless I’m probing properly?”. [DP1]*

This is something seen in both DPs working in GDS and PDS. An example from a dentist working in PDS demonstrates this:

*“We have … clinical portal which you can get a login for to look up the latest blood results which I would have access to but not every dentist would and then it’s to know whether it’s safe at that point from interpreting the blood results to actually go ahead and do the treatment so I personally wouldn’t always feel confident making that decision”. [DP7]*

One of the factors that appeared to strongly influence DP apprehension is the innovation in cancer care over the last few decades.

*“I suppose because I*,* I’m quite long in the tooth*,* shall we say. I’ve been graduated for 27 years and chemotherapy*,* radiotherapy*,* any cancer treatment has changed hugely in that time because when I graduated it was one treatment for all and now obviously they’re tailoring it as much*,* but the information and updates from oncologist to what we can and can’t do have changed massively”. [DP1]*

The participants working in the PDS often take referrals from General Dental Practitioners (GDPs) in GDS. In some cases, this apprehensiveness to treat could be ascribed to a lack of knowledge.

*“… [the GDP is] saying well*,* they’ve had radiotherapy for lungs so you know*,* we don’t know if we can take teeth out and it’s that being able to offer a reassurance to radiotherapy didn’t involve the jaws and the dentition and*,* and I guess the other big ones are … chemotherapy patients and when [the GDP] can intervene. So if somebody had chemotherapy six months ago*,* there’s absolutely no indication why they can’t safely manage teeth at that point if they’re not receiving regular blood bloods*,* or you know it’s cancer from a lymphoma from 1990*,* which there are still*,* you know*,* there are plenty of patients out there*,* it’s sort of saying it’s OK*,* don’t worry*,* you’re safe and you can you can manage that. [DP5]*

### Wider system factors influence management in primary care

Throughout the focus groups there were several factors raised which had an influence on the system that feed into many of the themes outlined in this study.

The role of secondary care appears to be tightly coupled to what happens in primary care. The pressures and capacity were mentioned on a few occasions but the role of guidance was hypothesised to potentially help with this by supporting patient management in primary care. Those working in hospitals and secondary care usually will have much more access to the patients’ medical information and oncology colleagues due to the digital systems in place. Continuity of care in PDS sites was also raised as an issue in some circumstances.

*“… PDS is under massive pressure at the moment and it’s not like we have available appointments and it usually means staff members being moved clinics or being held back to try and create space*,* or sometimes even other patients being moved to put in priority patients*,* so that I would say the most difficult things”. [DP7]*

*“But we’re very lucky in the PDS that we have got access to Trak and Portal [electronic patient records]*,* so it’s very easy for us to find out this information and … I think for GDPs some of them will be very motivated and will be keen to try and find out information from GPs some of them might not be so keen and they don’t have that network*,* so I think what might be a wee challenge”. [DP8]*

For DPs working in the PDS, access to GDPs was reported as a problem at the time this study was conducted. It meant that they felt unable to discharge some patients as there were scenarios where there was no GDP to discharge the patient to or an uncertainty if their patient would find a GDP.

The way dentists working under GDS regulations are paid in Scotland also has an influence on what can and cannot be provided.

*“… fluoride trays whether they are an item on the SDR [statement of dental remuneration] that needs to be sought for prior approval or whether that is something that GDP’s can provide*,* because that is something that could be a barrier to GDP’s offering that in general practice”. [DP5]*

There also appears to be variation in how patient charges are applied to oncology patients for GDS and PDS teams working under GDS regulations.

*“… one thing that never sat right with me was the fact that in [redacted]*,* we charged people for treatment*,* who were having to have things done because they were having cancer treatment*,* even though the referral came from a hospital I’ve always viewed it as part of their hospital treatment plan*,* they would have to pay for the extractions and things which I just thought was just the worst thing he had to then discuss with somebody but our new clinical director had said that [they] obviously looked into what’s going on in other health boards and we have now changed that so they’ll be treated without fee”. [DP7]*

When discussing the role of secondary care dental services for managing complex dental problems for oncology patients, some participants reported certain barriers in place. For those working in GDS, there was also a lack of awareness about the dental care pathways in place for patents with head and neck cancer. There were positive and negative experiences when it came to referring in patients with complex dental problems but there still appears to be some challenges regarding referral pathways into secondary care.

*“Have you ever tried to refer somebody to a dental hospital? It’s nigh impossible. [DP1]*

### Support

Participants were asked about sources of support when managing these patients. Throughout the focus groups, participants provided examples of areas where they felt they would need more support. Several of these have already been discussed. Several more examples emerged when participants were asked, ‘what topics would you like to see if new guidance was developed’ (Table 4). Participants in GDS were not aware of any oncology specific guidance but were aware of pre-existing SDCEP guidance that was relevant to aspects of oncology patients’ oral health care such as the Oral Health Management of Patients at Risk of Medication Related Osteonecrosis of the Jaw and the Management of Dental Patients Taking Anticoagulants or Antiplatelet Drugs [10, 20]. DPs working in PDS were aware of the Royal College of Surgeons guideline on Oral Management of Oncology Patients but some reported that they did not find it very user-friendly [4]. They also explained that specific guidance regarding the oral health management of oncology patients could help support them by reducing the level of unnecessary referrals made by colleagues in GDS. Participants all heralded SDCEP guidance and expressed a clear desire for similar guidance for the management of this group of patients.

*“So sort of having guidelines that*,* you know*,* primary care or GDP’s could follow I think that would be really good and just to give a basis of what can be managed*,* but then what would potentially need sort of referred on to more sort of specialist care areas would be really helpful”. [DP6]*

*“Yeah so I think*,* uh*,* an SDCEP guideline on this would be really helpful and I think I would echo what everyone else has said so far. The main thing I like about the guidance is how it’s laid out*,* it has a key points*,* the leaflets and it’s a bit of an easier read than for example*,* the BSDH guideline*,* which does have a lot of information and it is really helpful but perhaps the way it’s laid out isn’t particularly user friendly”. [DP4]*

A number of suggestions for topics to include in guidance and accompanying resources were made and are summarised in Table 5.

The utility of patient information in the form of leaflets was recognised and there appeared to be a desire for patient information on this topic.

Although many challenges were discussed during the focus groups, many participants did acknowledge that they worked with supportive colleagues in both dental and medical teams and that this support is invaluable.

**Table 5:** Summary of suggestions for guidance and accompanying resources

Suggestions for topics to include in guidance
Clear oral health messages and advice for dental and medical teams
Summary of oncology treatments and drugs and associated side effects
Dos and don’ts for dental examination and treatment
Treatment planning advice
**Suggestions for Accompanying Resources**
Patient information
Oncology ‘passport’ proforma which would contain the patient’s diagnosis, treatment and contact details for oncology team and used to aid communication
Photographs of side effects to look out for
List of advice DPs should provide to a patient pre-cancer treatment
Signposting to further support for patients (e.g. mental health advice) and further support for DPs (who and when to contact for advice)

## Discussion

This study provides an insight into the current practice and beliefs of primary care dental teams regarding the management of patients in primary dental care with, or a history of, a cancer. As far as the authors are aware, this is the first study to explore experiences of dental teams in their management of this cohort of patients. It does appear that this is a cohort of patients frequently seen in primary dental care settings. There is a wealth of literature on the oral health and dental management of patients with oral cancer; however, results from this study suggest that patients with other types of cancer are more frequently seen in primary care dental settings. Although there are guidelines and guidance available addressing this topic, the role of primary care dental teams is not clearly highlighted or defined. (3–4, 21, 22, 23) No participants working in GDS were aware of these guidelines when asked. They were, however, aware of other primary care focussed guidance such as those published by SDCEP. The Specialist Pharmacy Service provides advice on the management of patients during and following cancer treatment but does not include advice on pre-treatment [8]. Although guidance from the Royal College of Surgeons of England / The British Society for Disability and Oral Health and others exist, an apprehension about what can be safely provided in primary care was identified in this study and appeared to be one of the main reason patients are referred to secondary care dental services [4]. The challenges involved in treatment planning were also a main factor raised - similar to challenges in dental treatment planning prior to other surgical and medical treatments such as cardiac and transplant surgery or commencement of anti-resorptive and immunosuppressive agents.

Targeting further support and resource directly to dental teams only addresses a small part of the overall system. Concerns regarding the communication and mixed oral health messages between dental teams and medical teams require strategies to provide more consistency and more efficient and effective care for patients. Findings from the focus groups have highlighted several wider system factors, such as the interface between primary and secondary care, renumeration and access to dentists in primary care, that influence service provision and these will require macro system level consideration.

Importantly, the role and experience of patients was mentioned by participants throughout the focus groups. Exploration into the experiences of oncology patients and their oral health has not been previously addressed in the literature. Further research in this area is recommended to gain a fuller appreciation of the interaction between elements of the system.

The participants included in this study were self-selecting, thus a bias in the information provided is to be expected. By the nature of coming forward to participate, these DPs may have more experience and/or interest in treating this cohort of patients. DPs who have less experience may be underrepresented and thus the findings could be skewed.

Fifteen participants took part in the focus groups. The researchers were confident that data saturation was reached. However, it could be argued that a larger number of participants would have improved the validity and reliability of the results. Although invited to take part, it was not possible to recruit a dental nurse. The analysis was led by two dentists and again there could be bias in the way codes and themes were identified as they could be influenced by knowledge and previous experience. To mitigate this risk there were also members of the research team from non-clinical backgrounds involved in the analysis.

Further research will be required to validate the findings of this study as well as to explore the experiences of dental teams in other health systems. This study did not explore patient experiences and further research should also be targeted to address patient experiences of receiving oral health care when newly or previously diagnosed with cancer.

## Conclusions

This study has revealed experiences and challenges faced by dental teams in the provision of oral health care for patients with, or who have had, cancer. Results from this study could be used to help direct the development of further resources bespoke to primary care dental teams. They could also be used to inform where system improvements could be targeted to further support dental teams in their management of these patients. The findings from this study should be further validated by future research.

## Electronic supplementary material

Below is the link to the electronic supplementary material.

Supplementary Material 1

## Data Availability

The focus group questions can be found in the supplemental information provided. The transcripts from the focus groups are not available as participants did not consent for these to be made publicly available. Please contact the corresponding author for more information.

## Abbreviations

DCP: Dental Care Professional
DP: Dental Professional
GDP: General Dental Practitioner
GDS: General Dental Service
MDT: Multidisciplinary Team
MRONJ: Medication Related Osteonecrosis of the Jaw
NES: NHS Education for Scotland
NHS: National Health Service
NICE: National Institute for Health and Care Excellence
ORN: Osteoradionecrosis
PDS: Public Dental Service
SDCEP: Scottish Dental Clinical Effectiveness Programme
