# Supplementary material for: Experience of primary dental care teams in managing the oral health of oncology patients
Source: BMC Oral Health. 2024 Dec 26;24:1554. doi: 10.1186/s12903-024-05203-8 (PMC11673281; doi:10.1186/s12903-024-05203-8)
Supplement: Supplementary file 1 — Supplementary Material 1 [file 12903_2024_5203_MOESM1_ESM.docx]

**Experience of Primary Dental Care Teams in Managing the Oral Health of Oncology Patients: Additional File 1**

**Questioning Route / Strategy**

**Topic Questions in bold** and follow-up/prompting questions are bulleted underneath and will only be used if required.

**Intro to research**

Assume patient already has initial cancer diagnosis.

All stages of cancer treatment pre-, during-, post and palliative

**Ground Rules**

These will be confidential discussions.

Talking about cancer or your experiences of managing patients with cancer can be evoke emotion and could be upsetting to yourself and others.

With that in mind, please feel free to only answer questions you feel comfortable answering.

Please be mindful about the information you provide, respecting patient confidentially at all times.

If someone says something you agree with - we want to know.

If someone says something you don’t agree with - we want to know.

Reconfirm consent – opportunity to withdraw

Please use the raise hand feature if you want to speak.

Please use the chat to add any additional info

**---START RECORDING---**

**Participants to introduce themselves**

**Very briefly, what are the most common cancers in patients you see in your practice?**

- How many patients do you manage with a cancer diagnosis?
- Ask now if not mentioned:
- How many of these have an experience of Head and neck Cancer?
- How many patients are Children?
- Are many palliative?

**Quite briefly, what is your role in the management of these patients?**

- What is your role when a patient is diagnosed with a cancer?
- Is there any area where you are unclear on your role?
- What is your role with regards to prevention?
- Is your role different for different cancers?

**What are the roles of other members of the dental team in the management of these patients?** (depends on make-up of group  - only ask if no DCPs present)

**What do these patients present with when you see them?**

**(for check-ups, unscheduled care, assessment to ensure dentally fit?)**

**What are the challenges you face when seeing these patients?**

**What clinical aspect(s) do you feel you need more knowledge on when managing patients with an experience of cancer?**

- Possible Prompts – may want to explore:
- Awareness of cancer patients health – e.g. what can be safely treated in practice /is the patient healthy enough to treat (bloods etc).
- Knowledge around patients cancer treatment journey?
- What is appropriate for primary and what is appropriate for secondary / knowledge on referral pathways
- Managing treatment side effects – xerostomia, mucosits etc

- Is there a particular stage of their cancer treatment where you feel you deal with these patients most? (Pre/during/post treatment).
- Is there a time when management is most challenging

**What support is available when it comes to managing these patients?**

**Do you feel that the referral pathways for complex dental treatment are clear? (only ask this question if not mentioned in previous question)**

- Are you confident at knowing when to refer this group of patients?
- If not – why? What are barriers to referring to secondary care? What would increase confidence?
- Who do you refer these patients to, when required?
- What is your experience with the restorative consultant pathway for head and neck cancer patients?

- Who do you speak to for advice when required for these patients?

**What resources are available when it comes to managing these patients?**

- What guidance (if any) do you consult when managing these patients?

**Would you find specific guidance on the dental management of cancer patients in primary care useful?**

- What would the priorities be / main things that should be included?
- What would make a difference / improve things for you as a clinician
- What would make a difference / improve things for your patients?

**Any other comments / thoughts?**

- Happy to take follow-up thoughts via email

**---END RECORDING---**
